# Training the Armed Forces personnel in COVID-19 contact tracing: a Portuguese case study: Sofia Carmezim Pereira

**DOI:** 10.1093/eurpub/ckac131.096

**Published:** 2022-10-25

**Authors:** S Carmezim Pereira, A Bicho, I Guerra, M Borges, BS Vieira, D Loyens, F Barcelos, M Carreira, E Calé

**Affiliations:** Public Health Unit António Luz, ACES Amadora, Amadora, Portugal

## Abstract

**Background:**

Local contact tracing teams needed to be reinforced in preparation for a peak in Covid-19 cases. The Portuguese Armed Forces showed availability and their members initiated a formal training facilitated by the Public Health Unit (PHU) of Amadora. Health systems must be prepared to respond to all threats, as the COVID-19 pandemic showed us the need for quick task shifting and the training of non-experts’ workers.

**Objectives:**

The aim of the project was to develop contact tracing skills by non-health professionals, in the context of the COVID-19 pandemic. The training program was held online, with a total duration of 48 hours, distributed by the topics described: introduction to health and epidemiology concepts, the national guidelines, and the information systems (13h). We privileged demonstrative and participatory training methods, followed by continuous supervision of each contact tracing survey and constant feedback (35h). Learning support materials were sent out to assist the trainees, including written and video support.

**Results:**

More than 200 personnel - sailors, soldiers and airmen - were trained. Each Lisbon and Tagus Valley area PHU was reinforced with a team of military professionals in order to support the contact tracing process, with an increase in the number of surveys completed. We highlight as positive aspects: increased number of contact tracing surveys carried out; growth of inter-institutional partnerships; freeing up of specialized PHU resources to other important tasks. As for negative aspects we focus on the complexity in health communication, the limited time for training, and the lack of specific health knowledge of the trainees.

**Conclusions:**

This pandemic revealed the Portuguese need for a transdisciplinary approach in the provision of care, specially at a Public Health level. Training programs like these highlight the vital role of reshaping and reorganizing the healthcare workforce answering Public Health necessities.

**Key messages:**

• Training programs for non-health workers must be prepared to reinforce health systems when necessary. The reinforcement of contact tracing teams by the Portuguese Armed Forces was a great example.

• A transdisciplinary approach in the provision of care was essential during the COVID-19 pandemic. Specific training of non-health workers can be planned in time to respond to health threats.

